# Effect of Tibialis Anterior Neuromuscular Electrical Stimulation-Induced Eccentric Contraction Training on Single-Leg Standing: A Pilot Study

**DOI:** 10.3390/s25082455

**Published:** 2025-04-13

**Authors:** Nayoung Jeong, Doyeol Kim, Seonhong Hwang, Jongsang Son

**Affiliations:** 1 Department of Physical Therapy, Graduate School, Hoseo University, Asan 31499, Republic of Korea; nayoung0026@gmail.com (N.J.); 20192720@vision.hoseo.edu (D.K.); 2 Research Institute for Basic Sciences, Hoseo University, Asan 31499, Republic of Korea; 3 Smart Healthcare Convergence Research Center, Hoseo University, Asan 31499, Republic of Korea; 4 Department of Biomedical Engineering, Newark College of Engineering, New Jersey Institute of Technology, Newark, NJ 07102, USA

**Keywords:** neuromuscular electrical stimulation, tibialis anterior, ultrasonography, electromyography, dorsiflexion force, center of pressure, single-leg standing balance

## Abstract

This study explored the impact of a four-week Neuromuscular Electrical Stimulation (NMES)-induced eccentric contraction training on single-leg standing balance and muscle strength in 17 healthy adults. The unique training approach involved active antagonist muscle contraction during NMES. Post-training results revealed significant improvements in balance, with notable reductions in Center of Pressure (CoP) trajectory velocity (mean reduction: 0.07 ± 0.01 cm/s, *p* < 0.05) and range (mean reduction: 2.98 ± 0.53 cm, *p* < 0.05) on a firm surface. While increases in dorsiflexion force (mean increase: 21.43 ± 0.79 N, *p* < 0.05) and muscle activation were observed, these were not statistically significant. Changes in muscle pennation angles were also not significant (mean change: 0.43 ± 0.06 degrees, *p* > 0.05), underscoring the complexity of muscle adaptation processes. This study highlights NMES’s potential in enhancing balance and proprioceptive sensing, suggesting its promising applications in neuromuscular rehabilitation. However, further research is needed to fully understand its impact.

## 1. Introduction

Neuromuscular electrical stimulation (NMES) has been widely used in rehabilitation and sports medicine for inducing muscle contractions, effectively addressing muscle weakness and enhancing muscle strength [1,2]. Its applications extend to individuals with neuromuscular disabilities and progressive diseases, where voluntary muscle activation is limited [3,4]. Unlike voluntary contractions, which typically recruit motor units in order of size (small to large), NMES bypasses this principle and directly activates fast-twitch type II fibers [3,5]. This unique characteristic makes NMES particularly effective for improving muscle function, even in populations with compromised motor control [3,6]. Moreover, NMES is considered safe and easy to use, with minimal adverse effects reported in the literature [7].

Traditionally, NMES has been utilized to facilitate isometric and concentric contractions, primarily for strength development and rehabilitation [4,7,8,9,10,11,12]. However, recent studies have started exploring its potential for inducing eccentric contractions, a mode of muscle activation known for its superior effects on strength and hypertrophy [10,11]. Eccentric contractions occur when a muscle lengthens under tension, generating greater mechanical stress and leading to enhanced neuromuscular adaptations compared to concentric or isometric contractions [13,14,15,16]. The benefits of eccentric training are extensive, ranging from improvements in muscle strength and endurance to enhanced recovery from injuries [17,18,19]. Despite these advantages, voluntary eccentric training can be challenging due to its high force requirements and associated muscle damage risk [20,21]. This has led researchers to investigate alternative methods for facilitating eccentric training in a controlled and effective manner [14,22,23].

Recent evidence suggests that NMES can be used to induce eccentric contractions, providing a novel approach for training and rehabilitation [24,25,26]. However, most previous studies have relied on external mechanical systems to generate eccentric loading, which may not be practical for widespread clinical and athletic applications [27]. An alternative method involves leveraging voluntary antagonist contractions to induce passive eccentric contractions in the targeted muscle group [28,29]. This approach mimics natural eccentric movements while utilizing NMES to enhance muscle activation, making it a functionally relevant and accessible training method [9,30].

Single-leg standing balance is a fundamental component of functional mobility and serves as an important indicator of overall health and neuromuscular control [31,32]. The ability to maintain balance on one leg is essential for various daily activities and sports and is widely used as a predictor of fall risk and rehabilitation outcomes [33]. Assessing balance through single-leg standing tests provides critical insights into neuromuscular function, proprioception, and postural stability [34]. Advances in force plate technology and pressure sensor systems have significantly improved the accuracy of balance assessments by providing objective measurements of center of pressure (CoP) trajectories and postural sway [35,36]. These tools allow for the precise evaluation of subtle balance changes that may not be detectable through traditional clinical assessments [37,38].

The integration of NMES-induced eccentric contraction training with balance assessments remains largely unexplored in neuromuscular research [39]. While previous studies have demonstrated the benefits of NMES for strength and rehabilitation, as well as eccentric training for muscle adaptation, their combined effects on balance control remain poorly understood. In contrast, our study introduces a novel NMES-induced eccentric training method that does not require external mechanical assistance. Instead, it utilizes voluntary antagonist contractions to induce passive eccentric movements, mimicking eccentric exercises, which are often challenging to execute without assistance.

As a preliminary step, this study was conducted in healthy adults to first evaluate the feasibility and physiological effects of the training under controlled conditions, prior to application in clinical populations.

This study aims to evaluate the effects of a four-week NMES-induced eccentric contraction training program on single-leg standing balance, muscle strength, and neuromuscular activation. Specifically, we will assess balance performance (single-leg standing tests, CoP analysis), muscle strength (isometric and dynamic strength tests), and neuromuscular activation (surface EMG analysis).

We hypothesize that NMES-induced eccentric training will lead to improvements in postural control, as evidenced by a reduction in CoP trajectory velocity and range during single-leg standing, an increase in dorsiflexion maximum voluntary contraction (MVC) force, and potential changes in pennation angle and muscle activation, though the latter may require longer training durations to reach statistical significance. These outcomes are expected to provide new insights into the applicability of NMES-induced eccentric training for both rehabilitation and athletic performance.

## 2. Materials and Methods

### 2.1. Participants

This study involved a total of 17 participants (10 males and 7 females). The participants had an average age of 23 ± 1.41 years, a height of 168.88 ± 9.48 cm, and a weight of 66.45 ± 14.20 kg. This study received ethical approval from the Institutional Review Board of Hoseo University (1041231-221220-HR-157-01). All participants provided informed consent prior to the commencement of the test protocol. Seventeen able-bodied individuals (10 males, 7 females) volunteered to participate. The inclusion criteria were set as healthy adults with no lower limb injuries or pain, those who had not received electrical stimulation treatment on the anterior tibialis muscle within the last six months, and those comfortable with attaching electrodes to their skin. The exclusion criteria included individuals with a history of ankle joint or foot muscle injuries within the past year, those who had received electrical stimulation treatment on the anterior tibialis muscle within the last six months, and those who felt discomfort with attaching electrodes to their skin or with electrical stimulation.

### 2.2. Devices and Equipment

**Figure 1:**
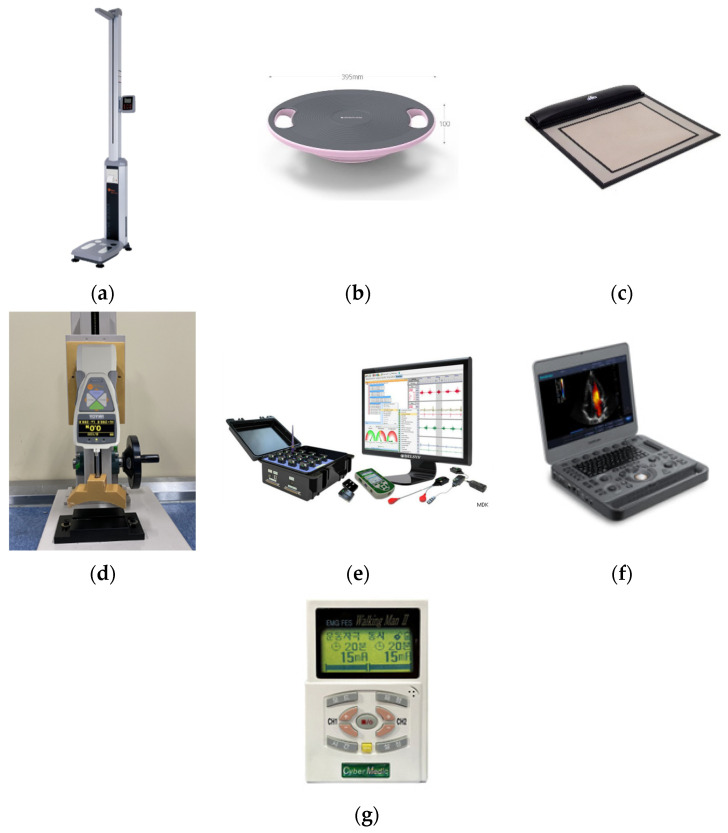
(**a**) A weight scale (GL-310P, GTECH Co., Ltd., Yangju-si, Republic of Korea); (**b**) a balance board (Egojin Ltd., Pocheon-si, Republic of Korea); (**c**) a pressure sensor system (MatScan VersaTek system, Tekscan, Inc., South Boston, MA, USA); (**d**) a push–pull gauge (ZTA-500N, IMADA Co., Ltd., Toyohashi, Japan); (**e**) a surface electromyogram (sEMG) sensor system (Trigno EMG sensor, Delsys, Inc., Natick, MA, USA); (**f**) an ultrasonography system (X5, SonoScape Medical Corp., Shenzhen, China); (**g**) a neuromuscular electrical stimulation system (EMS1000, Cybermedic Co., Ltd., Iksan-si, Republic of Korea).

A weight scale (GL-310P, GTECH Co., Ltd., Yangju-si, Republic of Korea) was used to measure subjects’ basic body weight and height (Figure 1a). A balance board (Balance board, Egojin Ltd., Pocheon-si, Republic of Korea) with dimensions of 395 × 395 × 100 mm^3^ (width × length × height), a weight of 1.3 kg, a maximum allowable load of 150 kg, and an inclination of 30 degrees was used for single-leg standing tests (Figure 1b).

A pressure sensor system (MatScan VersaTek system, TekScan, Inc., South Boston, MA, USA) and commercial software (FootMat Research 7.10, TekScan Inc., South Boston, MA, USA) were used to record the CoP trajectory when subjects performed single-leg standing (Figure 1c). The pressure mat hardware (Figure 1c) had sensing dimensions of 435.9 mm in width and 368.8 mm in height, with a spatial resolution of 0.014 sensors per square millimeter and a saturation pressure of 861.8 kPa.

A push–pull gauge (ZTA-500N, IMADA Co., Ltd., Toyohashi, Japan) was used to measure the ankle dorsiflexion force of subjects (Figure 1d). ZTA-500N is a force measurement sensor measuring 75 × 34 × 191 mm^3^ and weighing 490 g. It has a display resolution of 0.1 N (0.01 gf), an accuracy of ± 0.2%, and measurement ranges of ±500.0 N (50 kg). It supports USB and RS-232 analog outputs and software (Force Logger, IMADA Co., Ltd., Toyohashi, Japan) on a personal computer operating at 10 Hz. It offers a selection of measurement units (N and kg/g) and modes of regular, maximum, and negative measurement. In this study, we used ZTA-500N fixed to a manual handle-type gauge stand.

A surface electromyogram (sEMG) sensor system (Trigno EMG sensor, Delsys, Inc., Natick, MA, USA) and its commercial software (EMGWorks Acquisition & Analysis, version 4.7.3.0, Delsys, Inc., Natick, MA, USA) were used to record and analyze the muscle activation patterns of the tibialis anterior (TA) muscle (Figure 1e).

TA muscle architectures during voluntary and involuntary contractions were measured using an ultrasonography system (X5, SonoScape Medical Corp., Shenzhen, China). The transducer footprint size is 60 mm × 18 mm, and the center frequency is 7.5 MHz (Figure 1f). The transducer connector accommodates 3 probes, the monitor is 15.6 inches and high-definition (HD), the built-in battery supports 3 h of continuous scanning, and Wi-Fi and Bluetooth wireless connections are also available.

We used a neuromuscular electrical stimulation system (EMS1000, Cybermedic Co., Ltd., Iksan-si, Republic of Korea), attaching electrodes to the skin in an exercise stimulation mode to stimulate the TA muscle (Figure 1g). EMS1000 measures 130 × 86.5 × 30 cm^3^, uses a DC 9 V power adapter or a 9 V battery as a power source, and supports two channels for the simultaneous stimulation of two muscles. It has a stimulation frequency of 1 to 200 Hz, an output current of 0 to 99 mA, a duration of 0.5 to 30 s, a rest time of 0.5 to 50 s, a pulse width of 50 to 500 μs, ramp–up and ramp–down times of 0.5 to 10 s each, and output waveforms including monophasic, biphasic, and rectangular pulses.

### 2.3. Experimental Protocol

**Figure 2:**
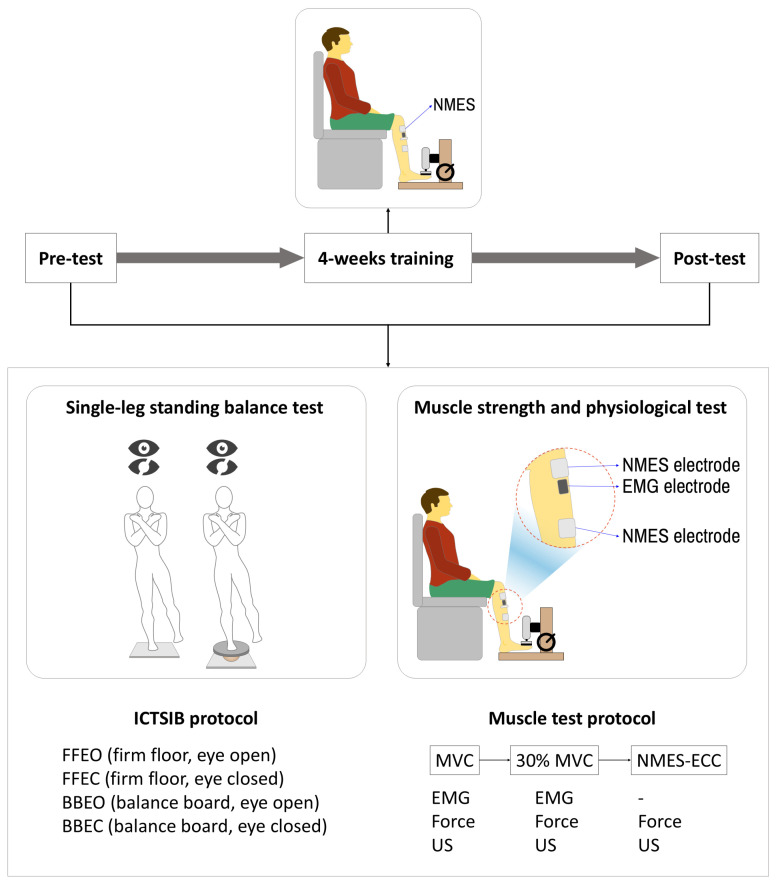
Schematic of the experimental protocol including balance and muscle testing before and after 4 weeks of NMES-induced eccentric contraction training.

**Figure 3:**
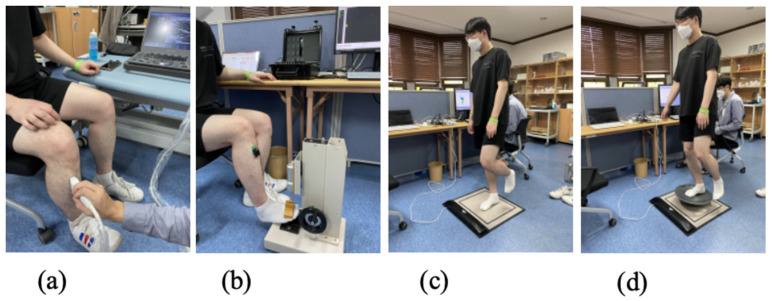
(**a**) Ultrasound imaging and measurements; (**b**) force and EMG measurements; (**c**) firm floor pressure sensor measurements; (**d**) balance board pressure sensor measurements.

After the interview and physical examination, NMES-induced eccentric contraction training was conducted over a period of four weeks. Pre- and post-tests, including single-leg balance tests and muscle physiology examinations—of strength, electromyography, and muscle structure (pennation angle)—were conducted before and after the four-week training period (Figure 2 and Figure 3).

All participants confirmed the absence of any structured exercise routines prior to the study, and were instructed to refrain from additional physical training beyond daily activities during the 4-week intervention period. This helped minimize variability due to baseline activity levels and allowed the observed training effects to be attributed more directly to the intervention itself. The details of each stage are as follows.

#### 2.3.1. Pre-Test

##### Single-Leg Balance Test

The single-leg balance tests for the subjects were conducted under four different conditions. Subjects performed a 30 s single-leg stance on a firm pressure mat sensor with eyes open (FFEO), on a firm pressure mat sensor with eyes closed (FFEC), on a balance board placed on the pressure mat sensor with eyes open (BBEO), and on a balance board with eyes closed (BBEC). During these tasks, the CoP trajectory was measured using the pressure mat sensor.

##### Muscle Physiology Examination

After the balance test, while the subject rested in a chair, the examiner attached NMES and electromyography electrodes to the subject’s TA and adjusted the height of the push–pull gauge stand lever so that the force sensor attachment lightly touched the dorsum of the foot. First, the maximum muscle contraction force and maximum electromyography of the subject’s TA were measured (3 s of maximum contraction followed by 3 s of rest, repeated 5 times). Then, after removing the electromyography electrodes, the ultrasound transducer was placed in the same spot to capture images of the TA muscle fibers during maximum contraction and relaxation using the same method. To ensure reproducibility and minimize measurement variability, the probe placement site was identified at one-third of the distance between the tibial tuberosity and the medial malleolus and marked using a semi-permanent skin marker. This standardized approach allowed for consistent transducer positioning across pre- and post-intervention assessments.

##### Determining NMES Intensity

After collecting muscle strength, electromyography, and ultrasound images, electrical stimulation was applied to the TA with the attached NMES electrodes to find the maximum intensity that caused dorsiflexion without discomfort and that was tolerable for the subject. At this point, NMES intensity and muscle strength were measured.

##### NMES-Induced Eccentric Contraction Training

All subjects underwent NMES-induced eccentric contraction training for 15 min per session, three times a week, for a total of four weeks, at the NMES intensity determined during the pre-test. The NMES stimulation intensity gradually increased and decreased to the set intensity with ramp up and down, and it was maintained at the set intensity for 3 s in a repeated manner. During this NMES to the TA, the subjects voluntarily contracted their ankle extensor muscles to induce passive eccentric contraction of the TA muscle. To minimize discomfort such as muscle fatigue and pain of the TA due to electrical stimulation, the training was conducted for 5 min with 1 min of rest, and the total stimulation time per session was around 15 min.

The NMES parameters were configured as follows: a stimulation frequency of 25 Hz [30], a pulse width of 300 μs [6], and a ramp–up and ramp–down duration of 2 s. These values were determined based on prior studies that demonstrated the effectiveness of low-frequency NMES for muscle strengthening and comfort, as well as the manufacturer’s guidelines for the stimulation device. The maximum stimulation intensity for each subject was set individually during a pre-test, corresponding to the highest current (within 20–40 mA) that did not induce pain or discomfort. The intensity range was selected in accordance with the device’s recommended operating limits. During the 4-week training period, the stimulation intensity was adjusted weekly by approximately 2% relative to the previous session, and participants were encouraged to train at the highest tolerable level without discomfort. This approach ensured both safety and consistency while accounting for individual variability in sensitivity and tolerance to electrical stimulation.

The NMES electrode placement followed the configuration shown in Figure 2, with electrodes attached to the proximal (knee-side) and distal (ankle-side) regions of the TA. The EMG electrodes were positioned immediately inferior to the proximal NMES electrode. During ultrasound imaging, the EMG electrodes were removed, and the ultrasound probe was placed in the same location to capture images of the TA muscle fibers. To ensure consistent electrode and probe placement across sessions, markings were drawn on the skin with a permanent marker and were retraced before each session to prevent fading.

To calculate 30% of Maximum Voluntary Isometric Contraction (MVIC), the EMG signal strength at the MVIC measured during the pre-test was used. A horizontal reference line representing 30% of the MVIC was displayed on the x-axis of the EMG measurement software version 4.7.3.0. During NMES-induced eccentric contraction training, subjects were instructed to monitor this display to ensure that their voluntary muscle activation remained at 30% of MVIC while undergoing NMES.

#### 2.3.2. Post-Test

The post-test was conducted in the same manner as the pre-test. Other notes during the test and training included allowing subjects to set the NMES intensity to their desired maximum, but once set, this intensity was maintained until the end of the four-week training. Subjects were also required to rest the day after training and perform the training three times a week, every other day. During the four weeks of training, subjects were asked not to perform any additional lower limb strength exercises like running or squats. The four-week training started within three days after the pre-test, and the post-test was conducted within three days after the end of the training. The areas where NMES and electromyography electrodes and the ultrasound transducer were attached or contacted were marked with a permanent marker to ensure they could be attached at the same location every time during training or measurement.

### 2.4. Data Processing and Analysis

#### 2.4.1. Single-Leg Stance Evaluation

To quantitatively evaluate the single-leg balance ability under four different conditions, variables were extracted from the measured CoP trajectory, including mean velocity (cm/s) in the medio-lateral (ML) and antero-posterior (AP) directions, maximum range (cm) in ML and AP, and planar deviation (cm) [40]. Additionally, the Romberg quotient was calculated to observe balance, which solely reflects proprioception contribution [41].

#### 2.4.2. Dorsiflexion (TA Muscle) Force

The dorsiflexion force of the foot measured at a sampling rate of 10 Hz from the push–pull gauge connected to a PC and the force during electrical stimulation were extracted as variables in Newtons for the maximum values.

#### 2.4.3. sEMG Signal Processing

**Figure 4:**
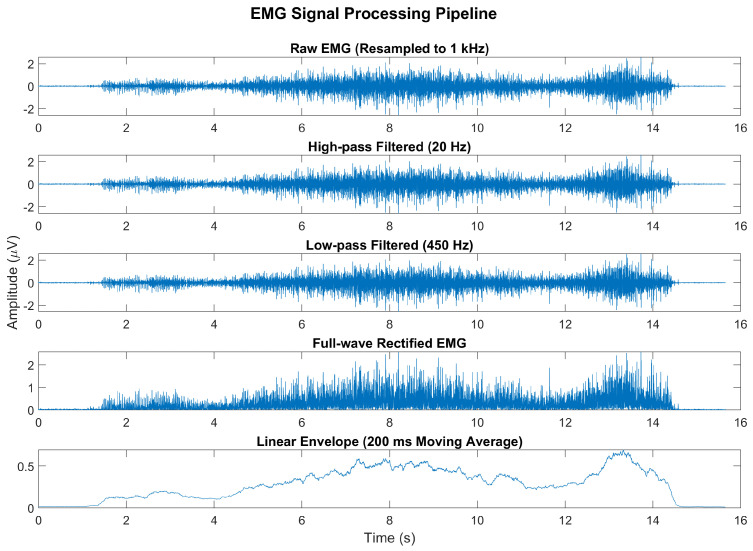
Representative example of the EMG signal processing pipeline. The raw EMG signal, originally sampled at 2 kHz, was resampled to 1 kHz and processed using a high-pass filter (20 Hz) and a low-pass filter (450 Hz). The signal was then full-wave-rectified and smoothed using a 200 ms moving average to produce a linear envelope of muscle activity.

The EMG signals were sampled at a frequency of 2 kHz and digitally re-sampled to 1 kHz prior to analysis. The signal processing pipeline included a high-pass filter with a cutoff frequency of 20 Hz to eliminate DC offset and low-frequency movement artifacts, followed by a low-pass filter at 450 Hz to remove high-frequency noise (Figure 4). After full-wave rectification, the signal was smoothed using a moving average filter with a 200 ms window to generate a linear envelope of muscle activity. All filters were 4th-order Butterworth filters. The signal was then normalized by the maximum voluntary contraction (MVC) of the TA.

#### 2.4.4. Pennation Angles of TA from the Ultrasound Images

Ultrasound images, captured using an X9 scanner (SonoScape, Shenzhen, China) with a linear probe (L742), were analyzed offline using the free NIH Image-J software (version 1.54g, NIH, Bethesda, MD, USA) for muscle fiber pennation angle computation [42]. Ultrasound image analysis was performed by one experienced researcher, and inter-observer reliability was assessed to ensure objectivity.

#### 2.4.5. Statistical Analysis

All data underwent normality testing (Kolmogorov–Smirnov test) and Levene’s test for homogeneity of variances before determining the method for non-parametric testing. For pre-post comparisons of variables extracted from the COP trajectory (such as velocity, range) and variables of muscle strength, electromyography, and pennation angles, the Wilcoxon signed-rank test, a non-parametric counterpart of the paired sample *t*-test (*α* = 0.05), was performed. Basic data handling and signal processing were conducted using Matlab (Matlab2021a, The Mathworks Inc., Natick, MA, USA), and all statistical tests were carried out using SPSS version 20.2 for Windows OS (IBM SPSS Inc., Chicago, IL, USA).

## 3. Results

This study involved a total of 17 participants (10 males and 7 females). The participants had an average age of 23 ± 1.41 years, a height of 168.88 ± 9.48 cm, and a weight of 66.45 ± 14.20 kg.

The NMES stimulation intensity was individualized for each participant during a pre-test session to ensure the maximum tolerable level without inducing pain. Initial stimulation intensities ranged from 22 mA to 39 mA, with male participants generally tolerating higher initial values than female participants. The training protocol adopted a progressive overload strategy, increasing the intensity by approximately 20% each week while maintaining a constant frequency of 25 Hz and a pulse width of 300 μs, in accordance with previous studies suggesting the efficacy of higher NMES intensities when tolerated [6,30,31].

Table 1 summarizes the weekly NMES intensities and percentage increases for each participant. While the overall pattern reflects a ~20% weekly increment, some participants exhibited slightly smaller increases during the third and fourth weeks due to comfort limitations approaching 60 mA. This gradual progression helped maximize training effects while respecting individual tolerance, consistent with recommendations to use intensities “as high as tolerable” to promote neuromuscular adaptation [6,31].

**Figure 5:**
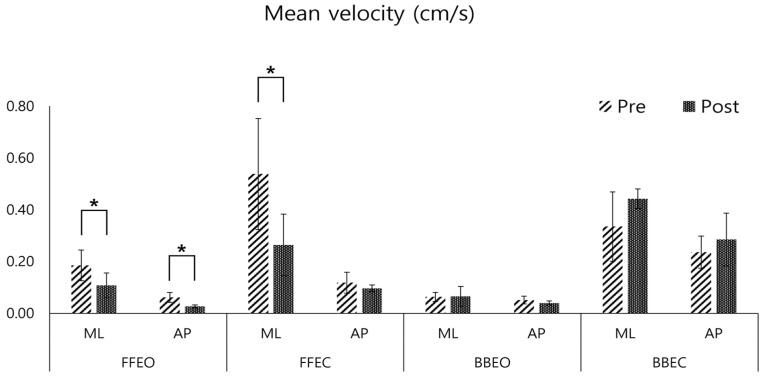
Pre- and post-comparison of the mean velocity of the center of pressure (COP) in the medio-lateral (ML) and antero-posterior (AP) directions under four conditions: firm floor with eye opened (FFEO), firm floor with eye closed (FFEC), balance board with eye opened (BBEO), balance board with eye closed (BBEC) (* indicates statistical significance).

In the post-training assessment, the velocity variables of the CoP trajectory showed a significant decrease in both the ML (mean reduction: 0.07 ± 0.01 cm/s, *p* < 0.05) and AP directions (mean reduction: 0.03 ± 0.01 cm/s, *p* < 0.05) when participants stood on a firm surface with eyes open (FFEO). In the FFEC condition, there was a significant decrease in ML direction velocity (mean reduction: 0.28 ± 0.09 cm/s, *p* < 0.05) (Figure 5).

**Figure 6:**
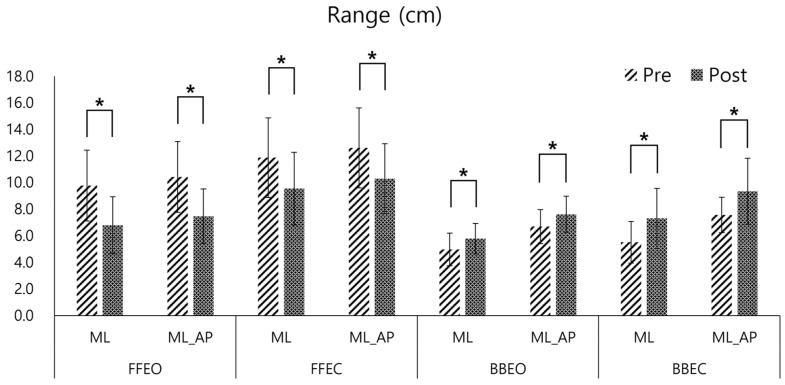
Pre- and post-comparison of the Center of Pressure (COP) range in the medio-lateral (ML) and Medio-lateral_antero-posterior (ML_AP) directions under four conditions: firm floor with eyes open (FFEO), firm floor with eyes closed (FFEC), balance board with eyes open (BBEO), and balance board with eyes closed (BBEC) (* indicates statistical significance).

The range variables of the CoP trajectory exhibited significant changes in the pre- and post-tests. On a firm surface (FFEO and FFEC), the range in both ML and AP directions decreased significantly after training (mean reduction: 2.98 ± 0.53 cm in ML FFEO, 2.96 ± 0.62 cm in AP FFEO, 2.33 ± 0.25 cm in ML FFEC, 2.31 ± 0.39 cm in AP FFEC, *p* < 0.05). Conversely, on the balance board, the range increased significantly (mean increase: 0.81 ± 0.07 cm in ML BBEO, 0.92 ± 0.08 cm in AP BBEO, 1.79 ± 0.07 cm in ML BBEC, 1.79 ± 1.16 cm in AP BBEC, *p* < 0.05) (Figure 6).

**Figure 7:**
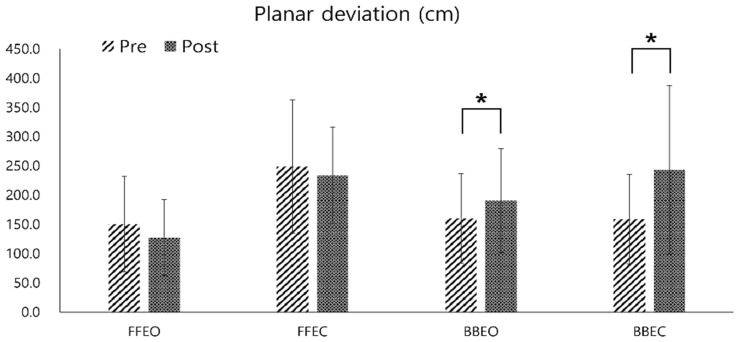
Pre- and post-comparison of the center of pressure (COP) planar deviation under four different conditions: firm floor with eyes open (FFEO), firm floor with eyes closed (FFEC), balance board with eyes open (BBEO), and balance board with eyes closed (BBEC) (* indicates statistical significance).

The planar deviation variable of the CoP trajectory showed a significant increase when participants were single-leg standing on the balance board post-training (mean increase: 30.19 ± 12.68 cm at BBEO, 84.81 ± 67.42 cm at BBEC, *p* < 0.05). However, the decrease in planar deviation on a firm surface post-training was not statistically significant (mean reduction: 22.94 ± 16.54 cm at FFEO, 15.17 ± 31.41 cm at FFEC) (Figure 7).

**Figure 8:**
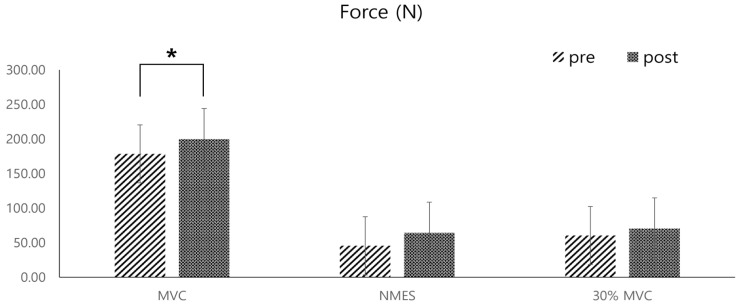
Pre- and post-comparisons of the average dorsiflexion forces of the ankle joint, measured by a push–pull gauge during three different types of dorsiflexion contractions: maximal voluntary contraction (MVC), passive contraction induced by NMES of the tibialis anterior muscle, and contraction at 30% of the MVC strength (* indicates statistical significance).

**Figure 9:**
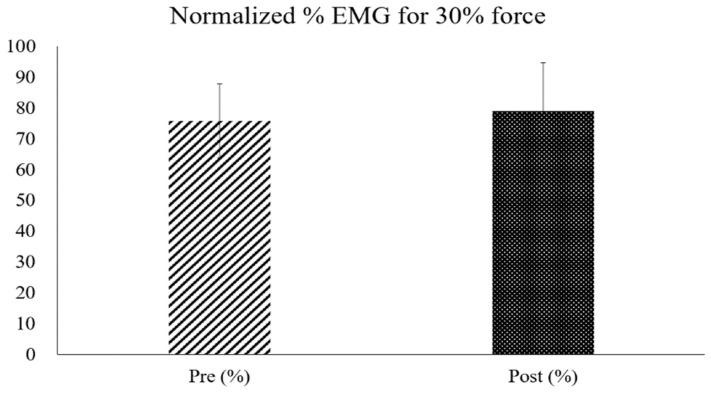
Comparison of pre- and post-test normalized EMG percentages measured during dorsiflexion at 30% of the MVC strength.

The average dorsiflexion force of all participants increased significantly in the MVC after the training (mean increase: 21.43 ± 0.79 N, *p* < 0.05) (Figure 8). However, the increase in maximum muscle strength measured during NMES and at 30% of the MVC muscle strength after the four-week training was not statistically significant (max strength increase during NMES: 18.74 ± 16.55 N, 30% MVC increase: 8.43 ± 9.99 N) (Figure 9).

**Figure 10:**
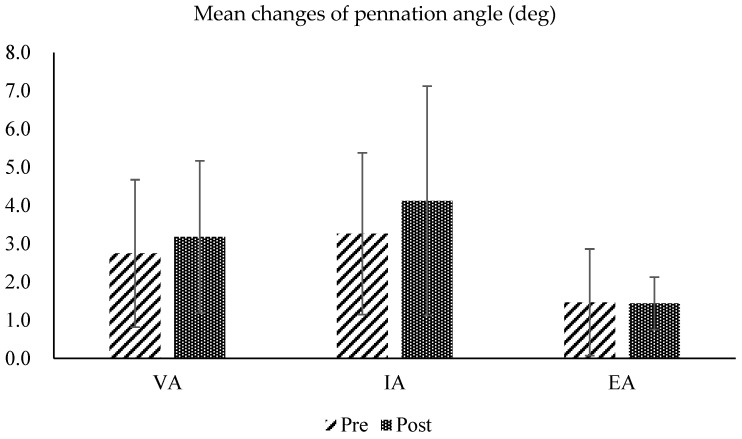
Pre- and post-test comparisons of the pennation angles calculated from ultrasound images of the tibialis anterior muscle, measured under three conditions: maximum voluntary contraction (voluntary activation: VA), passive contraction with NMES alone (involuntary activation; IA), and eccentric contraction during NMES with concurrent contraction of the ankle extensor muscles (eccentric activation; EA).

**Figure 11:**
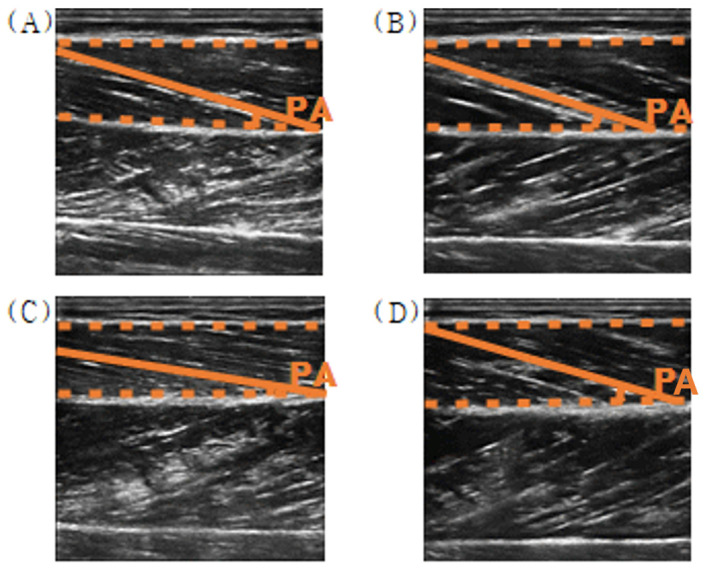
Example of the pennation angle in ultrasound images of the tibialis anterior muscle ((**A**) relaxed; (**B**) voluntary contraction, (**C**) involuntary contraction, and (**D**) eccentric contraction).

In the analysis of changes in the pennation angle of the tibialis anterior muscle fibers under three conditions, maximum voluntary contraction (voluntary activation: VA), passive contraction with NMES alone (involuntary activation; IA), and eccentric contraction during NMES with concurrent contraction of the ankle extensor muscles (eccentric activation; EA), there was a slight increase in both VA and IA in the post-test, but a slight decrease in EA. However, the changes in pennation angle under all three conditions were not statistically significant (mean pennation angle changes during VA: 0.43 ± 0.06 deg; IA: 0.86 ± 0.89 deg; EA: 0.03 ± 0.70 deg) (Figure 10 and Figure 11).

## 4. Discussion

This study represents an innovative exploration of the effects of NMES-induced eccentric contraction training, distinguishing itself from others through the novel use of NMES in a training regime that deviates from conventional methods. The unique approach of actively contracting the antagonist muscle during NMES-induced eccentric contraction, as opposed to relying on exoskeletal limbs, as in previous studies, offers a fresh perspective in neuromuscular training [37,38,43].

NMES-induced eccentric contraction training and voluntary eccentric contraction show significant differences in terms of the performance process, target of application, and purpose. First, there is a difference in the target of application and purpose. NMES-induced eccentric contraction training is mainly used for rehabilitation treatment to restore motor function in patients with neurological diseases such as stroke, whereas voluntary eccentric contraction is generally used to improve the functional exercise performance ability of healthy adults. NMES-induced eccentric contraction training is effective in improving muscle weakness or motor function decline due to nerve damage by quantitatively inducing the activation of specific muscles, whereas voluntary eccentric contraction is advantageous in implementing various movement patterns and improving functional movement. NMES-induced eccentric contraction training can be usefully utilized in clinical practice because it can provide a quantitative and systematic rehabilitation program for patients with neurological damage.

However, NMES-induced eccentric contraction training requires equipment and may lead to a difficulty in implementing natural movements during the contraction process. On the other hand, voluntary eccentric contraction has the disadvantage of requiring an initial motor learning process and having large inter-individual variability. Nonetheless, NMES-induced eccentric contraction training has a major advantage over voluntary eccentric contraction in that it can induce precise and quantitative muscle activation of the target muscle.

Previous studies have investigated NMES-induced eccentric contraction training in healthy adults; however, the effects of such interventions appear to be limited in this population [44,45,46,47]. Several key factors may account for this. First, healthy individuals typically possess intact neuromuscular control and relatively high baseline levels of muscle strength, resulting in a reduced potential for further improvement. Roig et al. (2009) reported that although eccentric and concentric resistance training improves strength in healthy adults, the effect sizes are generally smaller in high-functioning individuals [9]. Second, healthy participants are capable of performing eccentric contractions voluntarily, thereby diminishing the incremental benefits that NMES might provide. Supporting this, Da Silva et al. (2018) found no significant differences in strength gains between eccentric training alone and eccentric training combined with NMES in healthy subjects [39]. Third, because of their already optimized neuromuscular performance, healthy individuals may show limited neuromuscular adaptation in response to NMES interventions. Gondin et al. (2005) observed that NMES-induced improvements in neural drive and muscle structure are more prominent in individuals with lower baseline functional capacity [46].

In contrast, NMES-induced eccentric contraction training appears to be particularly beneficial for clinical populations such as individuals with stroke. Stroke often results in impaired voluntary motor control due to damage to the central nervous system. In such cases, NMES can bypass the compromised neural pathways and directly stimulate peripheral motor units, particularly fast-twitch type II fibers, thereby enabling eccentric contractions even in muscles with limited voluntary activation. This mechanism promotes neuromuscular re-education, enhances joint stability, and contributes to improved balance and functional recovery. Bortolotti et al. (2019) and McDowell et al. (2021) demonstrated, in systematic reviews, that NMES effectively improves muscle activation, reduces spasticity, and enhances overall functional outcomes in stroke rehabilitation [25,26]. Furthermore, Mettler et al. (2017) reported that NMES facilitates anabolic signaling pathways associated with muscle protein synthesis in stroke patients, providing physiological support for its rehabilitative effects [45].

While NMES-induced eccentric contraction training may offer benefits for both healthy individuals and clinical populations, its therapeutic impact is expected to be more pronounced in individuals with neuromuscular impairments, such as those recovering from stroke. Accordingly, the present findings provide a foundational rationale for future studies to explore the clinical applications of this training method in patient populations.

The training regimen, carried out over four weeks, was effective in enhancing single-leg balance abilities and muscle strength among participants. Significant decreases in the velocity, range, and planar deviation of the CoP trajectory on a firm surface (FFEO and FFEC conditions) were observed, suggesting an improvement in proprioceptive abilities essential for maintaining balance. This improvement in proprioceptive control is in line with findings from Lee, S.Y et al. [31] and Lehmann et al. [32], who emphasized the role of proprioception in balance stability. Conversely, the increase in CoP trajectory variables under the BBEC condition (balance board with eyes closed) after training demonstrates the challenges in balance maintenance without visual cues, highlighting the complex interaction between visual and proprioceptive inputs in balance control, as noted by Lindemann et al. [33] and Mike et al. [35].

This counterintuitive increase in CoP velocity following the intervention may be interpreted as a reflection of enhanced sensorimotor responsiveness under highly unstable and sensory-deprived conditions. According to Peterka [48], the central nervous system adapts to altered sensory environments through sensory reweighting, whereby reliance on remaining inputs—particularly proprioceptive and vestibular feedback—increases when visual information is unavailable. In this context, participants may have exhibited more active corrective sway as an adaptive strategy to maintain postural stability.

Additionally, it is possible that the intervention led to subtle changes in postural control strategies, enabling participants to engage in more dynamic balance adjustments rather than adopting overly rigid stances. Furthermore, the BBEC condition may have presented a task difficulty that exceeded the scope of functional adaptation achieved during the four-week training, potentially eliciting greater compensatory responses rather than reflecting a deterioration in balance control.

Analysis of the effect sizes (Hedges’ g) further supports these findings. CoP variables exhibited medium to large effect sizes, with the CoP mean velocity showing Hedges’ g values of 0.55 for the ML FFEO condition and 0.74 for the AP FFEO condition, and CoP mean range values exceeding 1.03 for both ML FFEO and ML_AP FFEO conditions. These results indicate that NMES-ECC training had a meaningful impact on balance capabilities. However, in contrast, the effect sizes for the force and pennation angle variables were below 0.5, indicating a limited impact on muscle strength and muscle architecture. This finding aligns with previous study [49]. Specifically, the force variable showed Hedges’ g values of −0.47 for MVC, −0.46 for NMES, and −0.42 for the 30% MVC condition, suggesting minimal improvement in muscle strength. Similarly, the pennation angle exhibited small effect sizes of −0.22 and −0.32 for the VA and IA conditions, indicating minimal changes in muscle structure (Table 2).

There was an increase in dorsiflexion force following NMES and at 30% of the MVC, although these changes were not statistically significant. However, a significant increase in MVC dorsiflexion force post-training indicates a positive effect of electrically stimulated eccentric contractions on maximum voluntary contraction strength. This finding aligns with the work of Reid et al. [24] and Roig et al. [9], who also reported improvements in muscle strength following NMES applications.

Regarding muscle activation and pennation angles, the study recorded slight increases post-training, but these changes were not statistically significant. This suggests that while NMES can influence muscle function, its effect on muscle architecture may require more prolonged training durations or different stimulation parameters. This interpretation is supported by previous studies reporting similar null effects in short-term NMES protocols [13,14,50]. It indicates that NMES’s role in structural muscle adaptations may be subtler and not as immediately apparent as its role in functional performance improvements.

The observed discrepancy between the significant increase in the MVC force and the non-significant change in pennation angle aligns with the well-established understanding that early-phase strength gains are largely driven by neural rather than structural adaptations. Short-term training, particularly when using NMES, often enhances motor unit recruitment, increases neural drive, and improves intermuscular coordination without inducing noticeable morphological changes. These findings are consistent with prior research suggesting that neural mechanisms predominantly account for early strength development following resistance training [7,46]. Structural changes such as increases in pennation angle or muscle hypertrophy typically require longer intervention periods or higher mechanical loading to manifest significantly.

In addition, this study has a limitation in that it was conducted only on an experimental group and lacked a control group, so significant changes between groups could not be confirmed. For future studies, a control group should comprise subjects that have not received training or a group of subjects who perform only voluntary eccentric contractions, without undergoing NMES-induced eccentric contraction training. Therefore, it is necessary to compare the experimental group with a group that performs ankle dorsiflexion and plantar flexion exercises using only voluntary eccentric contractions in future studies. In addition, it is necessary to confirm the actual changes between groups and confirm and compare the significance between the experimental group and the control group. Nevertheless, the improvements in postural control and dorsiflexion force observed in this study suggest that NMES-induced eccentric contraction training could be a feasible and effective strategy for improving balance in clinical populations, such as elderly individuals or patients with stroke. Given its simplicity and low-resource requirements, this method may be implemented in rehabilitation settings where access to advanced equipment is limited, enhancing patient accessibility to neuromuscular training.

## 5. Conclusions

In conclusion, this study demonstrated that four weeks of NMES-induced eccentric contraction training for dorsiflexor muscles in healthy adults led to improved balance abilities, indicating an enhancement in proprioceptive sensing. Although not statistically significant, there were indications of improvements in muscle strength, activation, and structure. The findings suggest that NMES-induced eccentric contraction training holds promise, particularly in the rehabilitation of patients with neuromuscular damage. This study highlights the potential of NMES in improving single-leg balance and muscle strength. However, the absence of significant changes in electromyography and pennation angles points to the complexity of muscle adaptation processes, emphasizing the necessity for further research to unravel these intricate interactions and to confirm the broader applicability of NMES-induced training in rehabilitation contexts.

## Figures and Tables

**Table 1:** Weekly NMES intensities and percentage increases over four weeks of training.

Subject No.	Sex	Weekly NMES Intensity				Intensity Increase (%)		
		1	2	3	4	2–1	3–2	4–3
1	M	31	37	44	53	19.4	18.9	20.5
2	M	28	34	41	49	21.4	20.6	19.5
3	M	37	44	53	60	18.9	20.5	13.2
4	M	39	47	56	60	20.5	19.1	7.1
5	M	35	42	50	60	20	19	20
6	M	32	38	46	55	18.8	21.1	19.6
7	M	37	44	53	60	18.9	20.5	13.2
8	M	29	35	42	50	20.7	20	19
9	M	31	37	44	53	19.4	18.9	20.5
10	M	34	41	49	59	20.6	19.5	20.4
11	F	22	26	31	37	18.2	19.2	19.4
12	F	26	31	37	44	19.2	19.4	18.9
13	F	30	36	43	52	20	19.4	20.9
14	F	30	36	43	52	20	19.4	20.9
15	F	27	32	38	46	18.5	18.8	21.1
16	F	24	29	35	42	20.8	20.7	20
17	F	23	28	34	41	21.7	21.4	20.6

Individual NMES intensity values (in mA) and week-to-week percentage increases for each participant. Stimulation intensities were adjusted weekly based on a ~20% increment from the previous week’s value, within the tolerable range for each subject.

**Table 2:** Effect sizes (Hedges’ g) for center of pressure (CoP) variables, force, and pennation angle across different conditions post-NMES-induced eccentric contraction training.

Variables	Condition	Hedges’ g	M_1_	M_2_	SD_pooled_
CoP mean velocity (cm/s)	ML FFEO	0.55	0.18	0.11	0.14
	AP FFEO	0.74	0.06	0.03	0.05
	ML FFEC	0.51	0.54	0.26	0.53
	AP FFEC	0.17	0.12	0.10	0.13
	ML BBEO	−0.03	0.06	0.07	0.09
	AP BBEO	0.15	0.05	0.04	0.07
	ML BBEC	−0.29	0.33	0.44	0.37
	AP BBEC	−0.19	0.24	0.29	0.25
COP mean range (cm)	ML FFEO	1.03	9.77	6.79	2.83
	ML_AP FFEO	1.04	10.42	7.46	2.79
	ML FFEC	0.74	11.87	9.54	3.09
	ML_AP FFEC	0.74	12.60	10.30	3.04
	ML BBEO	−0.64	4.97	5.78	1.25
	ML_AP BBEO	−0.65	6.68	7.60	1.39
	ML BBEC	−0.82	5.51	7.30	2.13
	ML_AP BBEC	−0.80	7.56	9.35	2.17
Planar deviation (cm)	FFEO	0.30	150.65	127.71	74.46
	FFEC	0.15	248.81	233.64	99.73
	BBEO	−0.35	160.43	190.62	83.94
	BBEC	−0.68	158.68	243.49	122.32
Force (N)	MVC	−0.47	178.54	199.97	44.70
	NMES	−0.46	45.57	64.32	39.53
	30% MVC	−0.42	24.93	33.36	19.65
Pennation angle (deg)	VA	−0.22	2.75	3.18	1.96
	IA	−0.32	3.26	4.12	2.61
	EA	0.02	1.47	1.44	1.09

## Data Availability

The data that support the findings of this study are available from the corresponding author upon reasonable request.
